# Implementing volunteer peer mentoring as a supplement to professional efforts in primary rehabilitation of persons with spinal cord injury

**DOI:** 10.1038/s41393-019-0294-0

**Published:** 2019-05-23

**Authors:** Dorthe D. Hoffmann, Joan Sundby, Fin Biering-Sørensen, Helge Kasch

**Affiliations:** 1 Spinal Cord Injury Center of Western Denmark, Viborg, Denmark; 2 Clinic for Spinal Cord Injuries, Rigshospitalet, Denmark; 3 University of Copenhagen, Copenhagen, Denmark; 4 Department. of Clinical Medicine, Aarhus University, Aarhus, Denmark

**Keywords:** Patient education, Spinal cord diseases

## Abstract

**Study design:**

A feasibility study, supplemented by a noncontrolled pretest/posttest.

**Objectives:**

To examine if a nationwide volunteer peer-mentoring program for in-patients with acute/subacute spinal cord injury is feasible and achievable.

**Setting:**

The Spinal Cord Injury Center of Western Denmark and Clinic for Spinal Cord Injuries, Rigshospitalet, Denmark.

**Methods:**

Volunteer mentor groups were formed similarly in two highly specialized SCI centers covering Denmark. Hospital staff was responsible for referral to the mentoring project and for the interdisciplinary evaluation of patient eligibility. At each of the two centers, a person living with the consequences of SCI coordinated the intervention in collaboration with healthcare personnel. Designated project personnel introduced eligible participants to the project. Staff at the SCI centers arranged to fit in the supplementary mentoring with ongoing treatment. A self-report questionnaire was completed prior to and after peer intervention. Outcome: patient reports regarding mentoring sessions, change in quality of life (QoL), depression items from the Short Form Health Survey (SF-36), pain frequency and intensity (11-point Numerical Rating Scale).

**Results:**

A nationwide mentor corps was established. Fifty-two eligible in-patients completed the study. Significant improvement in QoL was found after mentoring. Frequency and intensity of pain did not change, although five out of nine depression items improved significantly. A majority (94%) of the participants recommended others to meet with a peer mentor.

**Conclusions:**

Establishing a nationwide volunteer mentor system at a highly specialized neuro-rehabilitation units for SCI in-patients is both feasible and acceptable.

## Introduction

Spinal cord injury (SCI) is a relatively rare but life-altering condition, regardless of the extent and severity of the injury [1]. The world-wide incidence of SCI is estimated to be between 250,000 and 500,000 per year. Of the persons affected, 20–30% show clinically significant symptoms of depression, which is substantially higher than the general population [1, 2]. Persons with SCI score their quality of life (QoL) lower than the general population [3, 4]. Furthermore, the majority of persons with SCI suffer from pain [5], and they experience diminished participation in employment, recreation, and quality of interpersonal relationships in general [6, 7].

Several efforts are made to counter these issues through rehabilitation, described by WHO as a highly person oriented health strategy, consisting of “a set of interventions designed to optimize functioning and reduce disability in individuals with health conditions in interaction with their environment” [8]. The set of interventions in rehabilitation consists of many different options and combinations thereof largely depending on in which part of the world the rehabilitation takes place. One option is to include peers and their support and counseling in the variety of interventions in rehabilitation.

The concept “peer support” could be defined as providing assistance and encouragement by an individual considered equal [9], and “peer counseling” as a “combination of philosophical and methodological application of skills in basic human communication such as active listening, problem solving, resource identification and the support of a fellow human being” [10].

Healthcare services around the world include peer support as a significant element [9]. For example, in diabetes [11, 12] and cancer [13] rehabilitation, peer supported and even peer lead interventions are widely used in various forms, and they are often reported to have a positive and promising outcome; however, the scientific quality of evidence is low when it comes to determining which models and elements of peer support are best suited and most effective. In mental health services peer mentoring seems to have a positive influence on wellbeing in patients with mental disorders [14, 15] and in persons who have suffered a traumatic brain injury [16]. Recognizing the positive effects, specialist SCI rehabilitation centers also frequently provide peer support as an integral part of the service [10, 17]. In a randomized controlled trial [18] Gassaway et al. found that intensive one-to-one peer mentoring during and after SCI rehabilitation lead to greater gains in self-efficacy and to fewer days of rehospitalisation in the first 180 days after discharge.

Peer support and counseling come in many different forms and combinations: One-to-one or group-based, face-to-face or internet/telephone-based meetings, and peers as unpaid volunteers or as employed peer-workers. However, there is a lack of scientific evidence regarding the effectiveness of different forms of peer support in rehabilitation of persons with SCI [7].

Studies have shown that professionals can provide medical advice and information, whereas peers can be role models, and a supportive peer can be a powerful motivator and show by example that life after SCI can be meaningful [10]. Peers provide hope and make the newly injured person able to visualize his/her own potential for social participation [19]. Peer mentoring can be empowering and help newly injured towards coming to terms with their injury [17].

Peers are described as having credibility because “they have lived it all”; a living example or role model of what the SCI patients could achieve and a resource for how to achieve it [20]. People learn more and they try harder when they learn from others that they perceive to be like themselves, managing similar circumstances [18].

A common complaint of persons with neurological impairments is that their therapists know very little about living with disability, and thereby therapists are only providing little real-life experiential knowledge to the patients [21]. Furthermore, studies have shown that people with SCI need support from healthcare professionals, family, friends, and from other persons with SCI [22].

Denmark is a small country with 5.8 million inhabitants. Incidence of SCI is 130 per year [23, 24]. All primary rehabilitation after SCI in Denmark is concentrated at only two multidisciplinary and highly specialized rehabilitation centers, solely receiving patients diagnosed with spinal cord lesions. The two centers provide both in-patient hospitalization and out-patient clinics with life-long follow-up. From the acute or subacute phase at hospitals, patients are admitted to one of the two rehabilitation centers, depending on origin.

After discharge from primary rehabilitation, regular life-long out-patient clinic visits are offered. Readmission for in-patient stays at one of the centers is sometimes necessary. In Denmark healthcare is provided (including rehabilitation) free of charge for the individual.

A recent Danish study found that the major challenges of returning to one's own home after initial rehabilitation could be summarized into one term: “interpersonal relations”. For the individual person, leaving the rehabilitation center and peers it is difficult as you leave behind the persons that really understand what it means to live with an SCI [25]. Further research is needed to identify interventions that could enhance QoL during the early recovery and transition from SCI rehabilitation to home [26].

The purpose of this study was to investigate if organizing a nationwide cross-organizational peer mentoring system for in-patients with SCI in a primary rehabilitation as a supplement to high level professional neuro-rehabilitation would be feasible in a nationwide hospital setting and if it would be acceptable for patients to becoming mentees.

## Methods

The study was approved by the Danish Regional Ethics Committee, (journal number 2007-58-0010). The approval did not include access to electronic medical records.

Data were collected during a one-year inclusion period (1 January 2016 to 31 December 2016) in a joint venture between the SCI centers of Eastern and Western Denmark and RYK, the Danish Spinal Cord Injuries Association user organization.

### Study design and outcome measures

The design of this study was planned by a group of interdisciplinary professionals from the two rehabilitation centers and board members of the Danish Spinal Cord Injuries Association (RYK). The study was conducted as a feasibility study. Questionnaires were provided to mentees prior to the first and immediately after the last peer intervention. Our goal was to include 50 participants (i.e. mentees) in the project. The number of participants and the number of sessions were decided based on support obtained by funding made available for the study.

The following data were obtained through self-report questionnaires: Demographic data (age, gender, SCI level, cause of injury, social status, time since injury), QoL (International SCI QoL Basic Data Set [27] and selected parts of Short Form SF-36 v1 [28] (items 9a–9i) in a validated Danish version), pain score numeric rating scale (11-NRS) [29], and reported individual experiences with peer mentoring. We asked about the number of meetings and the satisfaction with the number of meetings. Furthermore, we asked whether mentees were satisfied with the mentor match that had been made for them.

The purpose of collecting data regarding QoL and pain was to monitor the participants’ health conditions during the intervention period.

SF-36 was chosen as a widely used as self-reported health-related outcome measure. SF-36 has shown to be applicable in SCI populations [30] and is recommended by The Spinal Cord Injury Rehabilitation Evidence Project as an objective measure of QoL in SCI [31]. Version 1 has been translated into many languages, including Danish. The SF-36 originally contains eight dimensions of health, but in this study only the part describing general health during the past 4 weeks was applied (items 9a–i). All questions posed to mentees in the study have been provided as supplementary material available for readers.

All nonparticipants (i.e. all patients that were in-patients but did not participate in mentoring sessions) were asked to answer a few questions about age, gender and if they had met, talked to, or participated in activities with one of the employed mentor coordinators. Finally, we asked which topics mentees discussed with the mentor coordinators. The nonparticipants answered the questionnaires within the last 2 days before discharge.

### Eligibility criteria

#### Mentees

Inclusion criteria: Persons with SCI, aged 18 years or more, who were primarily admitted to an in-patient hospital stay at a Danish SCI rehabilitation center and being able to understand written and verbal Danish at a sufficient level, were eligible to participate in the project.

Exclusion criteria: In order to ensure that mentors were not exposed to tasks and situations that would be very difficult to handle as nonprofessionals, patients with severe dementia, or other severe cognitive or psychiatric disorders (based on a conference between staff and project personnel) were excluded.

The interdisciplinary teams at the SCI centers were responsible for evaluating all newly admitted patients and their potential of participation in the project, evaluating fulfillment of in- and exclusion criteria and deciding when it would be feasible for the patient to receiving peer mentoring. Evaluations were based on subjective assessments made by the interdisciplinary teams in collaboration with project members.

Excluded patients were otherwise offered help by our skilled professionals such as psychologists, as a part of the highly specialized rehabilitation.

The designated project personnel introduced eligible participants to the project, and staff members at the SCI centers were informed of participation in order to fit in the supplementary mentoring with the ongoing standard care and rehabilitation.

#### Mentors

A nationwide corps of volunteer peer mentors was established. Some of the mentors were recruited from previously established peer-activities at the centers. Others were volunteers that contacted us at project start after information was provided at the Danish consumer organization's website (www.RYK.dk) and published in social media, such as Facebook^TM^. Mentors were required to possess a substantial (i.e. minimum of 2 years post SCI) experience of living with the consequences of SCI. They should be considered to having a settled view of their own situation and to be open-minded and tolerant. All these qualities were assessed by a conference between staff members and project group professionals, who had knowledge of the mentors’ current situation. This was possible, due a national life-long follow-up system with regularly recurring out-patient control visits at the out-patient clinic of the centers.

Before the mentoring sessions commenced, the mentors attended one introductory workshop each. Mentors were introduced to the following themes: ethics, legal conditions, confidentiality, and the role as volunteer peer mentor, i.e. what is expected from me? During the workshop, it was emphasized that peer mentors are not supposed to provide medical advice, whereas they were encouraged to share their personal experiences with the mentees.

The workshops lasted ~3 h and took place in groups at the two centers. The introductory workshop was repeated twice at each center in order to obtain complete participation. The charge nurses, psychologists, and the designated project personnel from the respective centers led the introduction. A few mentors, who were not able to participate in group workshops, were introduced individually and face-to-face by project personnel.

At subsequent meetings, organized by project personnel, mentors were invited to exchange their experiences amongst them. However, the requirement of maintaining the confidentiality regarding the mentees was underlined. If needed, professional support was available to all mentors throughout the project period.

All volunteer mentors worked as volunteers but were economically compensated for transportation costs.

#### Project personnel

At the two centers a total of four persons were employed part-time after they had applied for participation in the project. At each of the two centers a healthcare professional was employed for 5 h/week and a person with personal experience from SCI was employed with the tittle of mentor coordinator for 11 h/week. In conjunction, their job was (a) to introduce the project to eligible patients, (b) to match the mentees with suitable mentors, (c) to coordinate the mentor–mentee meetings, (d) to provide all mentees with questionnaires, and furthermore, and (e) to ensure that all practical tasks related to the project were solved.

#### Peer mentoring intervention

The mentor sessions were conducted as one-to-one meetings between mentor and mentee. Meetings could take place at the rehabilitation center (in a private room or in a more common area) or elsewhere if preferred. Meetings could occur anytime throughout the rehabilitation period, and the appropriate timing of initiating the sessions was based on a joint decision, made by the mentee, the interdisciplinary team, and the project members. There was no time limit for the duration of each meeting.

The participating mentees gave written and verbal consent of participation. Each participant was offered up to three formal (i.e. not incidental) sessions with a mentor as a start. In special cases, when it was considered necessary for the sake of the participants, project personnel could give permission to have more sessions. Mentors were individually chosen to match their specific mentees regarding age, gender, level of functioning or disability, vocational or leisure interests, or other more specific parameters as defined by the mentee. Mentees were asked to complete a questionnaire prior to the first mentor session and immediately after the final session took place. The questionnaires were short in order to keep the participants' load at a low level, ~60 questions before the first session and 50 questions after the last session.

### Statistics/data analysis

Data were analyzed with the software package STATA 15. (Texas, US™). Categorical data were reported as medians with 10th, 25th, 75th, and 90th percentiles (Box–Whisker plots). The sign-rank was used in paired comparisons or when considering more than two groups, the Kruskal–Wallis was applied for categorical data. With groups of less than five, Fischer's exact test was applied.

Data showing normal distribution after usual testing of normality (diagnostic Q plots, qnorm and use of histograms) were reported as means with standard deviations. Student's *t* test was applied in case of normal distribution, and *t* test values below 0.05 were considered significant.

SF-36 questions [32]: each item (from 9a–9i) was scored from: (1) All of the time, (2) Most of the time, (3) A good bit of the time, (4) Some of the time, (5) A little of the time, and (6) None of the time. In 9a, 9d, 9e, and 9 h obtaining lower scores was better, whereas in 9b, 9c, 9f, 9g, and 9i higher scores were preferable.

## Results

### Mentors

A nationwide corps of volunteer peer mentors was established. During a 1 year project period, 57 mentors were introduced to the program. Mentors were 37 men and 20 women, aged between 20 and 76 years. Of these, 34 mentors (22 men and 12 women) were involved in mentor sessions during the time-frame of the project. They were aged between 28 and 71 years. Mean age was of 49.4 years.

No mentors dropped out during the project period. Subsequently two mentors left the corps at their own request, due to personal matters.

Nineteen mentors had meetings with only one mentee, while 11 had meetings with two mentees, three had meetings with three mentees, and one had meetings with as much as five mentees. In three cases, one mentee had two consecutive mentors (one at a time).

### Mentees

Fifty-three patients were included as mentees in the project. (See Table 1 for further details).

**Table 1:** Study population characteristics

Characteristics	Menteés participating *n* = 52
Age at participation (median) (25th 75th percentile)	50 (34 58)
Years since injury (median) (25th 75th percentile)	0.34 (0.25 0.51)
Gender *n* (%)	
Men	33 (63.5)
Women	19 (36.5)
Spinal cord level *n* (%)	
Tetraplegia	15 (28.8)
Paraplegia	21 (40.4)
Missing	16 (30.8)
Cause of injury *n* (%)	
Trauma	23 (44.2)
Disease	29 (55.8)
Participants with pain *n* (%)	
Yes	39 (75.0)
No	13 (25.0)
Social status *n* (%)	
Married or companion	30 (57.7)
Live alone	15 (28.8)
Living with parents	3 (5.8)
Other	4 (7.7)
Children	
Yes	35 (67.3)
No	17 (32.7)

All 53 participated in mentor sessions. The data from one participant were subsequently withdrawn from the project due to protocol violation (did not fulfill inclusion criteria), leaving a total number of 52 participants (men: *n* = 33 (63%), women: *n* = 19 (37%)) aged between 19 and 77 years at participation. Self-reported data showed that 28.8% sustained a tetraplegia and 40.4% paraplegia, while almost a third (30.8%) did not answer this question. Traumatic cause was reported by 40.2% and 55.8% reported a nontraumatic origin of SCI. Two participants did not complete the follow-up questionnaire after the last mentor session.

**Fig. 1:**
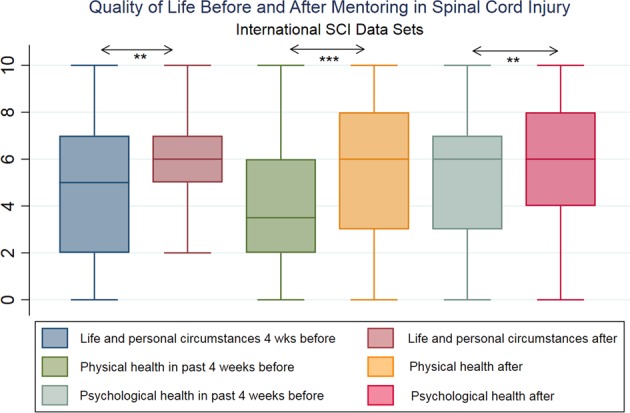
Quality of life before and after mentoring in spinal cord injury. International SCI data sets

Figure 1 shows a box-and whisker plot of the participants' QoL, rated before and after peer mentoring (International SCI QoL Basic Data Set [4]). At all three NRS-11-point scales, being Life and Personal Circumstances, Physical Health, and Psychological Health in past 4 weeks, participants gained significant improvement (KW, *p* = 0.01).

Reported pain frequency did not significantly change during the mentoring process, with 75–70% reporting ongoing pain (sign-rank, *p* = 0.48). The average 11-point NRS rating was a median 4 (25th; 75th 1; 5.5) prior to peer mentoring, and a median of 3 (25th; 75th 0; 6) post mentoring (signed-rank, *p* = 0.86).

SF-36 standard data showed significant improvement of depression-related items in five out of nine items. The remaining four items did not improve significantly, although the noted change in all cases was in a positive direction (see Table 2). In detail, *Did you feel full of pep* was improved, *Did you have a lot of energy* was improved, *Did you feel worn out* was improved, as was *Have you been a happy person* and *Did you feel tired*.

**Table 2:** Depression-related items (SF-36) scored by spinal cord injury in-patients before and after participation in a nationwide mentor program

SF-36 (During the past 4 weeks)	*N*	Score		Difference	Sign-rank
		Before	After	*Z*-value	*P*
*a) Did you feel full of pep?*	50	3.92	3.42	−3.45	0.00
b) Have you been a very nervous person?	*50*	*4.56*	*4.82*	*−1.26*	*0.21*
c) Have you felt so down in the dumps that nothing could cheer you up?	50	4.94	5.08	−1.22	0.22
*d) Have you felt calm and peaceful?*	*50*	*3.52*	*3.20*	*−1.74*	*0.08*
*e) Did you have a lot of energy?*	50	4.34	3.82	−2.74	0.01
f) Have you felt downhearted and blue?	*50*	*4.12*	*4.42*	*−1.94*	*0.05*
g) Did you feel worn out?	50	3.98	4.44	−2.16	0.03
*h) Have you been a happy person?*	*50*	*3.68*	*3.26*	*−2.27*	*0.02*
i) Did you feel tired?	50	3.30	3.78	−2.66	0.01

SF-36: Standard Form 36 questionnaire subscale applied

Scores ranged from: 1 All of the time, 2 Most of the time, 3 A good bit of the time, 4 Some of the time, 5 A little of the time, 6 None of the time

Italic rows are more positive with LOWER scores, non italic rows are more positive with HIGHER scores

*P  < 0.05

### Number of meetings with mentors

The number of meetings between mentee and mentor was registered. Of the 50 completing the final questionnaire, 11 (22%) reported that they had one meeting, 13 (26%) had two meetings, 19 (38%) had three meetings, and 7 (14%) reported more than three meetings. Thirty-two mentees (64%) found the number of meetings appropriate, 17 (34%) would have preferred further meetings. One mentee (2%) reported that he had too many meetings, and one did not answer. There was no correlation between the number of meetings and the reported satisfaction with the number of meetings. Details on non-participants as compared with participants are reported in tables 3 (demographics) and 4.

**Table 3:** Participants and non-participants in mentor–mentee project

	Participants (*N* = 52)		Non-Participants (*N* = 96)		*P*-value	
Gender (Percentage women (CI95))	19	37 (24; 51)	38	40 (30; 50)	NS	#)
Age (Median 25th 75th percentile)	50	(34; 58)	62	(49; 70)	*p* < 0.001	##)
First time admission (cases, percentage (CI95))	49	96 (87; 100)	57	59 (49; 69)	*p* < 0.001	#)
SCI Traumatic cases, percentage (CI95)	23	44 (30; 59)	36	38 (28; 48)	NS	#)

# Fischer's Exact/Chi2, ## Kruskal–Wallis

**Table 4:** In-hospital peer-mentor contact

	Participants (*N* = 52)			Non-participants (*N* = 96)			
	Yes	Percentage	(Ci95)	Yes	Percentage	(Ci95)	Fischer's exact
I have met the mentor contact	46	88	(77; 96)	60	63	(52; 72)	*p* < 0.001
I have talked with the mentor contact	35	67	(53; 80)	40	42	(32; 52)	NS
I have participated in mentor contact activities	12	23	(13; 37)	11	11	(6; 20)	NS
Subjects to discuss with mentor contact during stay							
We talked about disease/accident that caused my SCI	31	60	(45; 73)	16	17	(10; 26)	*p* < 0.001
We talked about my life during in-hospital rehabilitation	23	44	(30; 59)	12	13	(7; 21)	*p* < 0.001
We talked about my life afterwards the hospital	42	81	(67; 90)	18	19	(12; 28)	*p* < 0.001
We talked about my family	24	46	(32; 61)	10	10	(5; 18)	*p* < 0.001
We talked about my friends	16	31	(19; 45)	5	5	(2; 11)	*p* < 0.001
We talked about my work/education	24	46	(32; 61)	12	13	(7; 21)	*p* < 0.001
We talked about how to provide for oneself	8	15	(7; 28)	7	7	(3; 14)	NS
We talked about leasure activities, hobbies	24	46	(32; 61)	16	17	(10; 26)	*p* < 0.001
We talked about intimacy, sexuality	14	27	(16; 41)	2	2	(0; 7)	*p* < 0.001
We talked about bladder and bowel issues	21	40	(27; 55)	8	8	(4; 16)	*p* < 0.001
We talked about pain	20	38	(25; 53)	10	10	(5; 18)	*p* < 0.001
We talked about accessibility aids	32	62	(47; 75)	14	15	(8; 23)	*p* < 0.001
We talked about practical problems	38	73	(59; 84)	12	13	(7; 21)	*p* < 0.001
Other issues	11	21	(11; 35)	10	10	(5; 18)	NS
Importance of mentor contact during stay				27	28	(19; 38)	

### Topics discussed during mentoring sessions

The most frequently discussed topics were “life after hospitalization” (81%) and issues regarding practical problems associated with the mentees´ new situation (73%). See Table 4 for more information.

Satisfaction with peer mentoring Of the 50 participants answering the final questionnaire, 46 (94%) reported that they would recommend to meet with a peer mentor to other SCI in-patients, whereas 3 (6%) did not know, and one did not answer the particular question. No one (0%) would advice others not to meet with a peer mentor.

### Nonparticipants

Ninety-six persons (men: *n* = 58 (60%), women: *n* = 38 (40%)) completed the nonparticipant questionnaire. Of these, 59% were discharged from their initial rehabilitation. Table 4 shows difference in the topics approached by mentees with their mentors and nonparticipating SCI in-patients with the mentor contact, where much more subjects are discussed in the mentor–mentee relationship. Sixty-three percent of nonpartipants had met the mentor contact and 42% talked to him/her. Eleven percent had participated in mentor contact activities, and 28% of nonparticipants found it was important that mentor contacts were present at the hospital.

## Discussion

The main purpose of this study was to investigate whether it was feasible to organize a nationwide mentor corps and provide mentoring in highly specialized SCI rehabilitation hospitals. To our knowledge, this is the first nationwide study regarding peer mentoring among persons suffering from SCI. As there are only two SCI rehabilitation centers in Denmark and only a limited number of persons living with the consequences of SCI, it was possible to conduct and complete the project with a high degree of control and stringency. The establishment of a nationwide mentoring corps ensured that both SCI centers, despite the limited extent of mentoring, could benefit from the overall experience with resulting increase in the quality of work.

One task within the present study was recruiting persons that were suitable and qualified for being included in a corps of mentors. Not all individuals with SCI can be mentors [10]. Mentors do not necessarily need professional qualifications in order to share personal experiences and knowledge with newly injured persons, but some specific personal qualities are needed. Mentors should be empathetic and be able to listen, should be communicative and direct, should be sincere, ethical and trustworthy. Furthermore, they should have a broad range of positive characteristics, be comfortable with themselves, and open to others [10]. The above mentioned qualities could qualify you to be able to have a rewarding partnership with a newly injured and more inexperienced person, but several organizations recommend training to cope with the task [10] in order to improve the outcome. Since the study did not obtain data regarding the mentors' qualifications in our project, it is not possible to draw conclusions regarding e.g. correlations between mentors' qualifications and mentees' satisfaction with mentoring sessions.

When volunteer peers are involved in the rehabilitation process it is of importance to shield and protect them in their work. They should be prepared at best for the stressful situations that may arise during the sessions, and ongoing support should be available as requested. In this study all mentors were invited to an introductory workshop, in which they were given a brief introduction to selected topics, and they were invited to share their experiences within the whole mentor group at the centers. Furthermore, mentors were encouraged to seek help and support at the centers when needed. However, future studies should formally establish an ongoing support of mentors, as recommended by Chase [10].

In connection with the mentoring sessions was obtained self-reported data from the mentees. Pain was frequently encountered and of moderate intensity, however, no change in pain during the project period was found. Participating mentees reported significant improvement in both physical and psychological QoL dimensions. These findings correspond to those of Finnerup et al. [5] who reported slight but significant improvement of QoL during the first year after injury. Their study did not specify which efforts, in particular, made a contribution to improving QoL, so we do not know if peer mentoring was important for these observed improvements. However, QoL at least did obviously not deteriorate in the mentee as a result of peer mentoring. In addition, the SF-36 revealed a significant change for the better in six out of nine depression-related questions for study participants in the present study.

The number of meetings between mentees and mentors varied from one to three or more. More than half of the mentees were satisfied with the number, while a third answered that they would have liked more. There was no dose-response association, e.g. no correlation between the number of meetings, and the reported satisfaction with the number of meetings. One meeting could be beneficial, however, future studies should investigate the optimal number and extent of meetings. Intensive peer mentoring during and after rehabilitation have shown positive dose relationship with resulting increase of self-efficacy amongst mentees [18].

During the meetings between mentees and mentors, several topics were discussed. Most of the mentees (81%) reported that they had discussed their “life after hospitalization”. This is a very large and comprehensive topic that is not particularly specific, and presumably most mentees could recognize this topic in their mentoring sessions. Practical problems associated with the mentees' new life situation is more specific, and 73% discussed this issue. In order to prepare future peers and professionals for their work and to improve the quality of rehabilitation it would be helpful to more precisely investigating, which topics and issues the mentees need to discuss at a given time. This could be done in focus group interviews before and after peer mentoring participation.

After the mentoring sessions 94% reported that they would recommend others to meet with a peer mentor, and no one would recommend against it. The establishment is therefore considered widely accepted by participating SCI in-patients. Of three uncertain mentees regarding their recommendation, we have no further knowledge on this uncertainty. Recommendation should be further explored in future studies.

Data from the 96 nonparticipants did not differ from participants in distribution of age, gender. The nonparticipants reported which topics were discussed with the mentor coordinators. Some of the more intimate subjects like bladder and sexuality were not discussed here. Hobbies and leasure activities were discussed by some.

It would have been relevant to know the number of nonparticipants who were eligible to the project and contacted for participation, but had declined. We experienced that a few eligible patients did not want to participate in the project because participation entailed an expectation of completing two questionnaires. This was not documented, and in general the study failed to ask nonparticipants, if they would have met with a mentor if possible; however, only 28% of nonparticipants mentioned the present in-hospital mentor contact as of importance during their stay.

During the project period, project personnel with SCI were employed as mentor coordinators, and they were present at the centers for 11 h/week. After the project completion, both rehabilitation centers decided to prolong the employments for the two persons for a longer and not yet completed period. They have become essential assets as contact persons for patients and mentors and as the patients' representatives in the daily work at the centers. It is however arguable whether mentor coordinators become intramural “professionals” when time goes by. This issue needs further investigation.

## Conclusion

Organizing a nationwide volunteer mentor corps is feasible and peer mentoring during primary rehabilitation in highly specialized centers is feasible, accepted by rehabilitation staff and widely recommended by participating in-patients.

### Future directions and implications

The results of this study suggest that volunteer peer mentorship in primary rehabilitation of persons with SCI is a feasible and rewarding effort. The establishment of appropriate education programs in order to prepare SCI mentors for the task is needed. In a small country like Denmark, this should continue as a nationwide joint task.

The promising results of this feasibility study call for follow up randomized controlled studies. Focus interview studies could further substantiate findings from the present study. Peer mentoring studies using controlled designs could improve measuring of specific effects, e.g. self-efficacy, resilience and other extents of peer mentoring.

## Data archiving

The datasets generated and analyzed during the current study are available from the corresponding author on reasonable request.

## Supplementary information

Questionnaires 1 and 2
