# Supplementary material for: Implementing volunteer peer mentoring as a supplement to professional efforts in primary rehabilitation of persons with spinal cord injury
Source: Spinal Cord. 2019 May 23;57(10):881–9. doi: 10.1038/s41393-019-0294-0 (PMC6892415; doi:10.1038/s41393-019-0294-0)
Supplement: Supplementary file 1 — Questionnaires 1 and 2 [file 41393_2019_294_MOESM1_ESM.pdf]

Implementing volunteer peer mentoring as a supplement to professional efforts in primary rehabilitation of persons with spinal cord injury

Questions and response options – In Danish and translated into English

Spørgsmål	Svarmulighed	Question	Response option
**Spørgeskema 1 – før intervention**		**Questionnaire 1 – before intervention**	
Dato	Åbent tekstfelt	Date	Open text field
Køn	Mand/kvinde	Gender	Male/female
Fødselsdato	Åbent tekstfelt	Date of birth	Open text field
Hvornår fik du din rygmarvsskade	Åbent tekstfelt	When did you sustain your injury	Open text field
Hvornår blev du indlagt på KRS/VCR	Åbent tekstfelt	When were you admitted to the rehabilitation center	Open text field
Er dette din første indlæggelse på KRS/VCR	Ja/nej	Is this your first admission to the SCI rehabilitation center	Yes/no
Hvad er årsagen til din rygmarvsskade	Sygdom/traume	What was the cause of your spinal cord injury	Disease/trauma
Hvad er dit skadesniveau<br>(Skriv gerne AIS score)	Åbent tekstfelt	What is the level of your injury<br>(Please write the AIS score if possible)	Open text field
Hvordan er din familiære situation lige nu	• Gift eller bor med partner<br>• Bor alene<br>• Bor hos forældre<br>• Andet	How is your family situation right now	• Married or living with partner<br>• Living alone<br>• Living with parents<br>• Other
Har du børn	Ja/nej	Do you have children	Yes/no
Hvor stor en del af tiden i de sidste 4 uger har du<br>• følt dig veloplagt og fuld af liv<br>• været meget nervøs<br>• været så langt nede, at intet kunne opmuntre dig<br>• følt dig rolig og afslappet<br>• følt dig fuld af energi<br>• følt dig trist til mode<br>• følt dig udslidt<br>• været glad og tilfreds<br>• følt dig træt	• Hele tiden<br>• Det meste af tiden<br>• En hel del af tiden<br>• Noget af tiden<br>• Lidt af tiden<br>• På intet tidspunkt	How much of the time during the past 4 weeks<br>• did you feel full of pep<br>• have you been a very nervous person<br>• have you felt so down in the dumps that nothing could cheer you up<br>• have you felt calm and peaceful<br>• did you have a lot of energy<br>• have you felt downhearted and blue<br>• did you feel worn out<br>• have you been a happy person<br>• did you feel tired	• All of the time<br>• Most of the time<br>• A good bit of the time<br>• Some of the time<br>• A little of the time<br>• None of the time
Når du tænker på dit liv og dine personlige omstændigheder, hvor tilfreds har du været med dit liv de sidste 4 uger	0-10 skala	Thinking about your own life and personal circumstances in the past four weeks, how satisfied are you with your life as a whole	0-10 numeric rating scale
Hvor tilfreds er du med dit fysiske helbred de sidste 4 uger	0-10 skala	How satisfied are you with your physical health in the past four weeks	0-10 numeric rating scale
Hvor tilfreds er du med dit psykiske helbred, følelser og humør de sidste 4	0-10 skala	How satisfied are you with your psychological health, emotions and	0-10 numeric rating scale

Questions and response options – In Danish and translated into English

**Spørgsmål**	**Svarmulighed**	**Question**	**Response option**
uger		mood in the past four weeks	
Har du smerter<br>Hvis ja:<br>• På en skala fra 0-10, hvordan vil du beskrive din smerte<br>• Hvordan vurderer du din smerte, når den er mindst intens<br>• Hvordan vurderer du din gennemsnitlige smerte gennem de seneste 2 døgn<br>• Hvordan vurderer du din smerte lige nu	Ja/nej<br><br>0-10 skala	Do you have pain<br>If yes:<br>• On a 0-10 scale, how will you rate your pain<br>• How is your pain, when it is least intense<br>• How do you rate your average pain over the last 2 days<br>• How do you rate your pain right now	Yes/no<br><br>0-10 numeric rating scale
Hvem fortalte dig først om mentorordningen	• Personalet<br>• En folder<br>• Opslag<br>• En anden patient<br>• Hjemmeside<br>• Mentorkontaktpersonen opsøgte mig<br>• En anden mentor<br>• Andet	From where did you first hear about the mentoring	• Staff<br>• A leaflet<br>• A poster<br>• A fellow patient<br>• A website<br>• Project personnel came to me<br>• A mentor came to me<br>• Other
Hvad ønsker du, at du og din mentor kommer til at tale om	• Sygdommen eller ulykken der var årsag til min rygmarvsskade<br>• Livet som patient under indlæggelsen<br>• livet efter udskrivelse<br>• Familie<br>• Venner<br>• Arbejde/uddannelse<br>• Forsørgelse<br>• Fritidsinteresser<br>• Sex og samliv<br>• Blære- og tarmfunktion<br>• Smerter<br>• Hjælpemidler<br>• Praktiske problemer<br>• Andet	Which issues do you wish/want to talk to your mentor about	• Trauma or disease that caused my injury<br>• My life during hospitalization<br>• My life after hospitalization<br>• Family<br>• Friends<br>• Work and education<br>• My economy<br>• Leisure activities<br>• Sexuality<br>• Bladder and bowel function<br>• Pain<br>• Aids and equipment<br>• Practical problems in general<br>• Other
Tror du at det kan være en fordel for dig i nogle situationer at tale med en mentor frem for at tale med en	Ja/nej/ved ikke	Do you think it might be an advantage for you in some situations to talk to a mentor instead of a professional staff	Yes/no/do not know

Questions and response options – In Danish and translated into English

Spørgsmål	Svarmulighed	Question	Response option
fagperson		member	
Tror du at det kan være en fordel for dig i nogle situationer at tale med en mentor frem for at tale med din familie eller venner	Ja/nej/ved ikke	Do you think it might be an advantage for you in some situations to talk to a mentor instead of a member of your family or friends	Yes/no/do not know
Hvad håber og/eller forventer du at få ud af at deltage i mentorordningen	Åbent tekstfelt	What do you hope for / expect to get out of meeting with a mentor?	Open text field
Har du nogen kommentarer	Åbent tekstfelt	Do you have any comments	Open text field
**Spørgeskema 2 – efter intervention**		**Questionnaire 2 – after intervention**	
Dato	Åbent tekstfelt	Date	Open text field
Hvor længe har du været indlagt på KRS/VCR	Åbent tekstfelt	For how long have you been at the rehabilitation center	Open text field
Hvordan er din familiære situation lige nu	• Gift eller bor med partner<br>• Bor alene<br>• Bor hos forældre<br>• Andet	How is your family situation right now	• Married or living with partner<br>• Living alone<br>• Living with parents<br>• Other
Hvor stor en del af tiden i de sidste 4 uger har du<br>• følt dig veloplagt og fuld af liv<br>• været meget nervøs<br>• været så langt nede, at intet kunne opmuntre dig<br>• følt dig rolig og afslappet<br>• følt dig fuld af energi<br>• følt dig trist til mode<br>• følt dig udslidt<br>• været glad og tilfreds<br>• følt dig træt	• Hele tiden<br>• Det meste af tiden<br>• En hel del af tiden<br>• Noget af tiden<br>• Lidt af tiden<br>• På intet tidspunkt	How much of the time during the past 4 weeks<br>• did you feel full of pep<br>• have you been a very nervous person<br>• have you felt so down in the dumps that nothing could cheer you up<br>• have you felt calm and peaceful<br>• did you have a lot of energy<br>• have you felt downhearted and blue<br>• did you feel worn out<br>• have you been a happy person<br>did you feel tired	• All of the time<br>• Most of the time<br>• A good bit of the time<br>• Some of the time<br>• A little of the time<br>• None of the time
Når du tænker på dit liv og dine personlige omstændigheder, hvor tilfreds har du været med dit liv de sidste 4 uger	0-10 skala	Thinking about your own life and personal circumstances in the past four weeks, how satisfied are you with your life as a whole	0-10 numeric rating scale
Hvor tilfreds er du med dit fysiske helbred de sidste 4 uger	0-10 skala	How satisfied are you with your physical health in the past four weeks	0-10 numeric rating scale
Hvor tilfreds er du med dit psykiske helbred, følelser og humør de sidste 4 uger	0-10 skala	How satisfied are you with your psychological health, emotions and mood in the past four weeks	0-10 numeric rating scale

Questions and response options – In Danish and translated into English

Spørgsmål	Svarmulighed	Question	Response option
Har du smerter<br>Hvis ja:<br>• På en skala fra 0-10, hvordan vil du beskrive din smerte<br>• Hvordan vurderer du din smerte, når den er mindst intens<br>• Hvordan vurderer du din gennemsnitlige smerte gennem de seneste 2 døgn<br>• Hvordan vurderer du din smerte lige nu	Ja/nej<br><br>0-10 skala	Do you have pain<br>If yes:<br>• On a 0-10 scale, how will you rate your pain<br>• How is your pain, when it is least intense<br>• How do you rate your average pain over the last 2 days<br>• How do you rate your pain right now	Yes/no<br><br>0-10 numeric rating scale
Hvor mange gange har du haft møde med din mentor	• 1 gang<br>• 2 gange<br>• 3 gange<br>• Andet	How many sessions have you had with your mentor	• One<br>• Two<br>• Three<br>• Other
Mener du, at det var et passende antal møder	• Ja, det var passende<br>• Nej, det var for få møder<br>• Nej, det var for mange møder	Do you think that the number of meetings was appropriate	• Yes, it was appropriate<br>• No, it was too few meetings<br>• No, it was too many meetings
Hvor mødtes I	• På centeret<br>• Andet	Where did you meet	• At the rehabilitation center<br>• Other
Hvor lang tid varede møderne<br>(Svar for hvert møde)	• Under 1 time<br>• Ca. 1 time<br>• 1-2 timer<br>• Mere end 2 timer	How long did the meetings last<br>(report for each meeting)	• Less than an hour<br>• Approximately one hour<br>• Between one and two hours<br>• More than two hours
Hvad var det vigtigste, du og din mentor talte om	• Sygdommen eller ulykken der var årsag til min rygmarvsskade<br>• Livet som patient under indlæggelsen<br>• Livet efter udskrivelse<br>• Familie<br>• Venner<br>• Arbejde/uddannelse<br>• Forsørgelse<br>• Fritidsinteresser<br>• Sex og samliv<br>• Blære- og tarmfunktion<br>• Smerter<br>• Hjælpemidler<br>• Praktiske problemer	What was the most important issues, that you and your mentor discussed	• Trauma or disease that caused my injury<br>• My life during hospitalization<br>• My life after hospitalization<br>• Family<br>• Friends<br>• Work and education<br>• My economy<br>• Leisure activities<br>• Sexuality<br>• Bladder and bowel function<br>• Pain<br>• Aids and equipment<br>• Practical problems in general<br>• Other

Questions and response options – In Danish and translated into English

Spørgsmål	Svarmulighed	Question	Response option
	• Andet		
Var det en fordel for dig i nogle situationer at tale med en mentor frem for at tale med en fagperson	Ja/nej/ved ikke	Was it an advantage for you in some situations to talk to a mentor instead of a professional staff member	Yes/no/do not know
Var det en fordel for dig i nogle situationer at tale med en mentor frem for at tale med din familie eller venner	Ja/nej/ved ikke	Was it an advantage for you in some situations to talk to a mentor instead of a member of your family or friends	Yes/no/do not know
Var den mentor, som blev valgt til dig, den rette til at indgå i samtaler med dig i forhold til de emner, du ønskede at tale om	Ja/nej	Was the mentor that was selected for you the right one to talk to, regarding the issues that you wanted to talk about	Yes/no
Har du haft den samme mentor gennem hele forløbet	• Ja<br>• Nej, jeg bad om at få en anden mentor<br>• Nej, jeg fik en anden uden selv at have bedt om det	Have you had the same mentor throughout the process	• Yes<br>• No, I asked for another mentor<br>• No, I was given another mentor without having asked for it
Har du oplevet at møde den mentor-kontaktperson, der er ansat på KRS/VCR, mens du har været indlagt<br><br>Hvis ja: i hvilken sammenhæng mødte du kontaktpersonen	Ja/nej<br><br>• Jeg har bare set ham/hende<br>• Jeg har talt med ham/hende<br>• Jeg har deltaget i aktiviteter med ham/hende	Have you experienced meeting the mentor contact person, who is employed at the rehabilitation center, during your hospitalization<br><br>If yes: How did you meet	Yes/no<br><br>• I just saw him/her<br>• I talked to him/her<br>• I participated in some activities with him/her
Skriv med egne ord, hvad du har fået ud af at deltage i mentorordningen	Åbent tekstfelt	Write in your own words what you have gained from participating in the mentoring program	Open text field
Vil du anbefale andre at tage imod tilbud om at tale med en mentor	Ja/nej/ved ikke	Would you recommend others to meet with a mentor	Yes/no/do not know
Har du nogen kommentarer	Åbent tekstfelt	Do you have any comments	Open text field
